# Anesthetic implications for transurethral resection of prostate in a COVID-19 survivor with Stanford-A aortic dissection with acute urinary retention due to metastatic carcinoma prostate: a case report

**DOI:** 10.1186/s42077-022-00296-1

**Published:** 2023-01-02

**Authors:** Shagun Bhatia Shah, Itee Chowdhury, Venkatesh Pally, Chamound Rai Jain

**Affiliations:** Rajiv Gandhi Cancer Institute and Research Centre, Sec-5, Rohini, Delhi 110085 India

**Keywords:** Aortic dissection, COVID-19, Carcinoma prostate, Labetalol, Subarachnoid block

## Abstract

**Background:**

Aortic dissection is a new addition to the long COVID-19 complication catalog. We report this rare and novel complication, which can be missed without a high index of suspicion in the ever-burgeoning population of COVID-survivors presenting for un-related surgery. We emphasize the importance of recording blood pressure in both the upper limbs in COVID-survivors during pre-anesthetic checkup, especially in patients with a dilated aorta on the chest radiograph to identify any interarm blood pressure discrepancy characteristic of aortic dissection. Discontinuation of antihypertensive based on low/normal blood pressure in left upper limb can precipitate concealed and catastrophic rise in blood pressure in the right upper-limb propagating the dissection of aorta to a fatal conclusion. The cardinal anesthetic consideration is to mitigate the effect of hemodynamic perturbations on the dissected aorta.

**Case presentation:**

We report the successful management of the case of a 76-year-old male prostatic cancer patient with COVID-induced aortic-dissection and acute urinary retention, posted for transurethral resection of prostate. CT angiography revealed an intimal flap in the ascending aortic lumen and aortic arch till the origin of left subclavian artery resulting in a double-barreled aorta. An arterial line was secured in right radial artery and non-invasive blood pressure recorded in left arm simultaneously (202/60 mmHg in right upper-limb and 92/70 mmHg in the left upper-limb on wheeling into the operation theatre). He underwent transurethral prostatic resection and bilateral orchidectomy under low-dose subarachnoid block with prophylactic use of labetalol infusion.

**Conclusions:**

The importance of recording blood pressure in both the upper limbs in COVID survivors maintaining a high index of suspicion for aortic dissection cannot be overemphasized. Transurethral prostatic resection surgery under low-dose subarachnoid block is possible under the umbrella of judicious selection and optimal use of cardiac medication with an interventional cardiologist as standby in patients with aortic dissection.

## Background

The incidence of acute thoracic aortic dissection is 2.53/100,000/year (Melvinsdottir et al. 2016). An aortic intimal tear that allows blood to dissect between the tunica media and intima of the aortic wall maybe multifactorial in origin (hypertension, connective tissue disorders (Marfan’s Syndrome; Loeys-Dietz syndrome), idiopathic, iatrogenic, smoking, cocaine-abuse) (Gregory et al. 2018). Coronavirus disease 2019 (COVID-19) has been implicated with unpredictable cardiovascular involvement (cardiac insufficiency, myocarditis, arrhythmias stemming from hydroxychloroquine, and other antiviral drugs) (Clerkin et al. 2020; Shah et al. 2021). Our case study is unique because firstly, it highlights a new potential complication of COVID-19 namely aortic dissection; secondly, it emphasizes that transurethral resection of prostate under subarachnoid block is possible in aortic dissection patients; and thirdly, it describes in detail how beta blockers (especially labetalol) are the cornerstone of preoperative, intraoperative, and postoperative management of such patients with inputs from an interventional cardiologist.

DeBakey (types I, II, IIIA, IIIB) and Stanford (types A and B) based on the site of dissection are the traditional classifications while the Penn classification based on localized (organ malperfusion/branch vessel occlusion) or generalized (circulatory collapse) ischemic symptoms at presentation predicts mortality after aortic dissection repair (Gregory et al. 2018). Amongst patients with acute Stanford type-A aortic dissection, 48.6% die before reaching hospital and 30-day mortality rate is 47.4% in survivors to hospital admission. Subsequent 5-year survival rate is high (85.7%) in the 27% patients who survive (Melvinsdottir et al. 2016).

## Case presentation

**Fig. 1:**
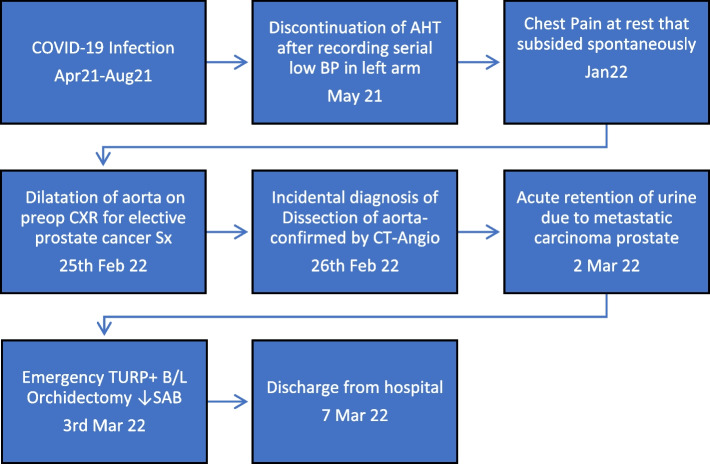
Timeline of events (AHT, antihypertensives; BP, blood pressure; CXR, chest radiograph; SAB, subarachnoid block; TURP, transurethral resection of prostate)

Our patient was a 76-year-old male weighing 69 kg with metastatic carcinoma prostate (American Joint Committee on Cancer Stage-IVb) with acute urinary retention, posted for transurethral resection of prostate and bilateral orchidectomy. After testing COVID-positive by the reverse transcriptase polymerase chain reaction (RTPCR-test), 10 m prior to surgery, he had received a full course of paracetamol 650 mg, apixaban 10 mg, ivermectin, and steroids. His fever although high-grade initially was mild for the following 4 months (Fig. 1). He tested negative for typhoid and tuberculosis.

He was a chronic hypertensive on amlodipine (5 mg) and prazocin (5 mg) combination, intermittently for the last 10 years, and completely off-treatment for the ensuing 5 months post-COVID attributable to consistently low blood pressure recorded in his left upper limb. During the pre-anesthetic check-up, 104/70 mmHg was the non-invasive blood pressure (NIBP) recorded in the left upper limb. Telmisartan 80 mg was prescribed elsewhere before he presented at our institution. He gave a history of chest pain 2 months back while relaxing in his armchair, which was sudden in onset, spread to the back, and subsided spontaneously. Since his offspring were settled abroad, the patient refrained from bothering them unduly with his health concerns which delayed the diagnosis. The patient had a negative history of surgical procedures, cardiac catheterization, congenital connective tissue disorders, and cocaine abuse.

Computerized tomographic angiography revealed an intimal flap in the ascending aortic lumen and aortic arch till the origin of left subclavian artery resulting in formation of a double-barreled aorta with a large false lumen and small true lumen. The descending thoracic aorta and the abdominal aorta were spared. The right and left coronary arteries could be seen arising from the right and left coronary cusp which was adequately supplied by the true lumen. The false lumen supplied the right brachiocephalic trunk and left common carotid artery (LCCA). An atherosclerotic plaque was precariously perched in the aortic arch and extended into the brachiocephalic trunk. The ascending aorta was dilated. The flap extended into the proximal part of LCCA. The false lumen appeared thrombosed. The flap measured 3.5 cm from the aortic annulus and 1 cm from the sinotubular junction (Stanford Type A dissection).

**Fig. 2:**
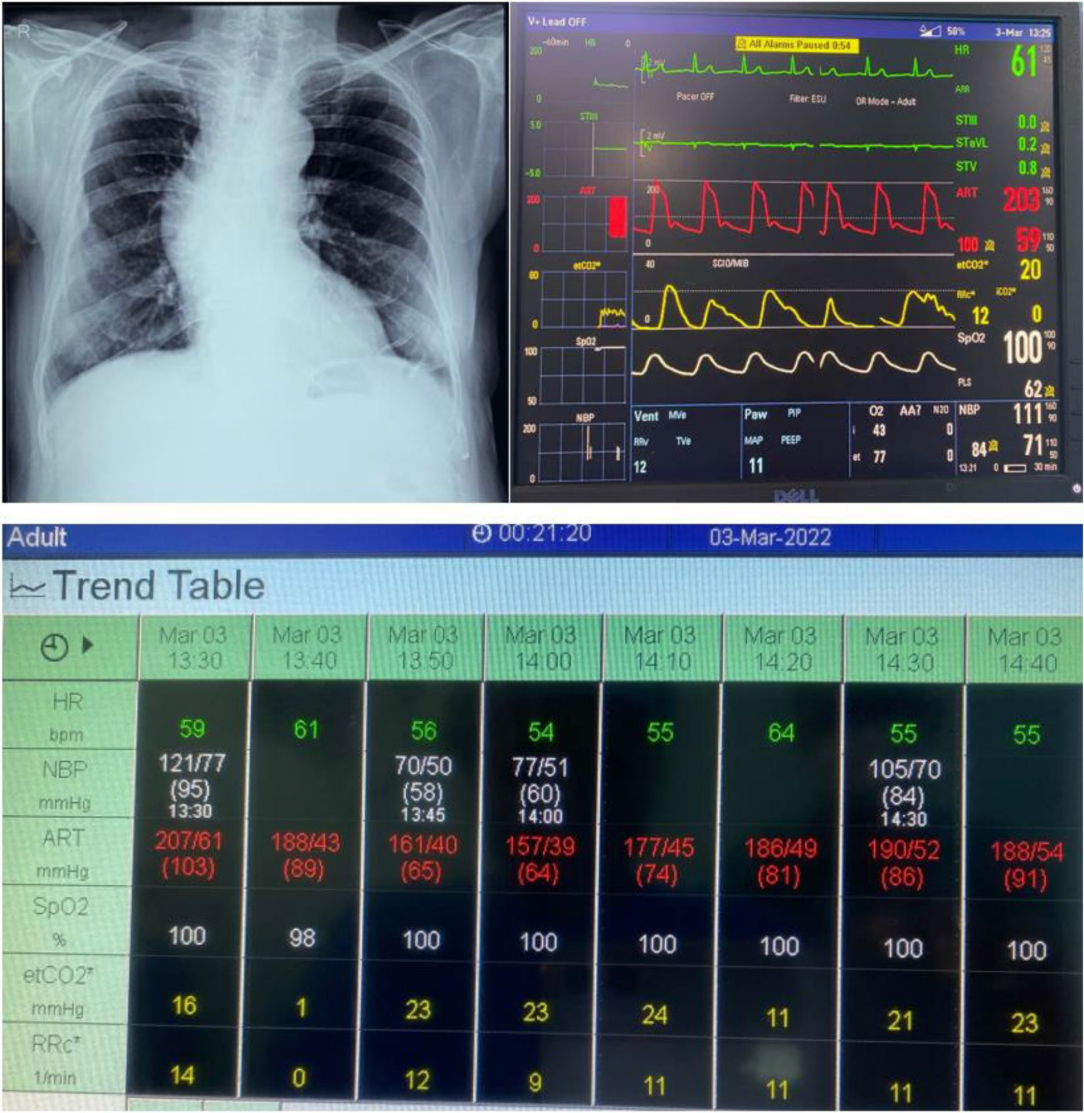
Chest radiograph with dilatation of the aorta (upper left); Monitor screen showing the disparity in blood pressures between both the upper limbs (upper right); Trend table depicting the intraoperative hemodynamic changes over time (bottom row)

Chest radiograph showed fibrotic changes in left lower lung lobe, besides a significantly enlarged aorta (Fig. 2). Echocardiography revealed a left ventricular ejection fraction (LVEF) of 60%. On re-checking the BP in light of CT angiographic findings, 165/72 mmHg was the non-invasive BP in the right upper limb while that in the left upper limb was 108/69 mmHg.

The patient was orally administered labetalol 50 mg (9AM and 9PM) 1 day prior to surgery and at 6:30AM on the morning of surgery.

After application of standard monitors in the operation theater, the blood pressure in the right upper limb was 202/60 mmHg on right radial artery cannulation but that in the left upper limb was 92/70 mmHg at the same time point. IV labetalol was administered in four 5 mg boluses followed by an infusion of labetalol 1 mg/ml @ 2–6 ml/h (started in the OT and continued for 8–10 h in the postoperative period) under supervision of a senior interventional cardiologist.

Subarachnoid block was instituted in L3-4 interspace, in sitting position by midline approach using a 27-G Quinke needle with 2.4 ml 0.5% heavy bupivacaine. The BP came down to 162/56 mmHg 5 min later. After 70 min, there was a progressive rise in invasive blood pressure (IBP) despite increasing the labetalol infusion rate and three midazolam boluses of 0.25 mg each intraoperatively. The IBP had spurted to 215/76 mmHg when the patient was shifted to the surgical intensive care unit after completion of surgery at 95 min post-subarachnoid block. The patient was continuously monitored for features of TURP syndrome, but serial arterial blood analysis (ABG) (including serum sodium) was within normal limits. There was no chest/back pain throughout the procedure. Proximal urethral stricture, grade 3 enlarged prostate (6.5 (craniocaudal) × 7.5 (anteroposterior) × 5.7 (transverse) cm with bladder-neck mass invading verumontanum, bilateral vesicouretric junctions not visible even after resection were the findings that made it an emergency surgery. The patient was switched to oral labetalol 100 mg twice a day after 8 h. The blood pressure was 160/70 mmHg on the second postoperative day and 142/66 mmHg on discharge 3 days later. Tight blood pressure control and serial aortic imaging surveillance post-discharge was recommended.

## Discussion

Inflammatory and acute phase reactant-induced damage, immune-mediated injury, augmented sympathetic activity or therapeutic drugs are contributory factors to aortic dissection in a patient with influenza and coronavirus disease (Ashur et al. 2020). Two case reports about aortic dissection diagnosed in COVID-positive patients have been reported till date (Tabaghi et al. 2020; Fukuhara et al. 2020).

A decision to proceed with the surgery was taken since firstly it was an emergency surgery and secondly because the 5-year survival rate is high in the 27% patients who survive beyond 30 days after acute aortic dissection. The two principles during anesthetic management are firstly, avoiding tachycardia and secondly, avoiding hypertension. Tachycardia augments the shear stress due to pulsations while hypertension increases the pressure on the intimal walls of the aorta. Both potentially lead to progression of aortic dissection and aortic rupture (Totonchi et al. 2015). Hence, to avoid hemodynamic perturbations/alpine anesthesia due anesthetic induction and intubation, regional anesthesia was preferred over general anesthesia. Aiming to reduce the force of the left ventricular contraction without compromising perfusion, nitroglycerine being a venodilator has a scant role in the management of patients with aortic dissection. In these patients, the ideal initial step is a beta-blocker (esmolol/metoprolol) to reduce the heat rate to acceptable levels (60/min) followed by an arteriodilator like sodium nitroprusside to reduce the systolic blood pressure to 110–120 mmHg (Hebballi and Swanevelder 2009). Beta-blockers (esmolol, metoprolol, labetalol) should precede vasodilators, to avoid potential increase in left ventricular contractions due to reflex catecholamine release due to vasodilatation. Calcium channel blockers are useful in patients where beta-blockers are contraindicated (Erbel et al. 2001). Avoid inotropes, hydralazine (potentially increases aortic wall sheer stress) vasodilation before beta blockade (causes reflex sympathetic activation) and pericardiocentesis in tamponade (causes exsanguination). Hemodynamic goals include preload maintenance without aggressive fluid therapy which may worsen dissection, heart rate < 60 bpm with beta blockade, normal sinus rhythm, and reduction in contractility with beta blockade to reduce sheer stress on intima (Hiratzka et al. 2010). Our patient was administered labetalol (combined alpha–beta adrenergic antagonist). Labetalol lowers blood pressure by decreasing systemic vascular resistance by α1-blockade and at the same time counteracts the reflex tachycardia from vasodilation through its β-blocker effect, hence preferred.

The discontinuation of antihypertensive therapy in the post-COVID period stemming from a false low recording of NIBP in the left upper limb led to a spike in the already high right upper limb BP augmenting aortic dissection. The BP recorded in the right upper limb should be considered as the true BP in these patients. The double-barreled aorta with a narrow patent barrel presented two distinct suggestive clinical features. Firstly, a wide pulse pressure (difference between systolic and diastolic blood pressures) in the right upper limb and secondly a large pressure differential between the two upper limbs. Since the right and left coronary arteries were supplied by the true lumen a myocardial infarction did not ensue and LVEF was maintained.

Endovascular interventions aimed at reconstruction of the aortic segment harboring the intimal tear, induction of thrombosis of the false lumen, and re-establishment of the true lumen patency and side branch flow were not advised in our patient by the interventional radiologist since there was a pre-existing fortuitous thrombosis in the false lumen at the time of diagnosis of aortic dissection preoperatively.

Uncontrolled essential hypertension with erratic treatment was the cause of intraoperative hypertension. Although we momentarily considered TURP syndrome, it was dismissed by absence of hyponatremia on ABG and because the rule of 60 (duration of TURP < 60 min; height of glycine/irrigation fluid bag < 60 cm) was not breached and there was no chest/back pain throughout the procedure.

Strengths of our approach comprise meticulous history taking, physical examination, and a battery of radiological and biochemical investigations to elicit the etiology of aortic dissection and possible association with COVID-19, research on optimal cardiac and anesthetic medication to minimize shear stress on aorta, and an experienced interventional cardiologist as standby during surgery.

Limitations to our approach include the absence of a standby cardiothoracic surgeon and extracorporeal membrane oxygenation facility to cover the worst possible scenario of progression of dissection and a ruptured aorta.

## Conclusions

A high index of suspicion for aortic dissection should arise in COVID-survivors with enlarged aorta on routine chest radiograph. BP should be recorded in both upper limbs in such patients. Emergency surgery under subarachnoid block is possible under the umbrella of judicious selection and optimal use of cardiac medication with an interventional cardiologist on standby.

## Data Availability

The datasets used and/or analyzed during the current study are available from the corresponding author on reasonable request.

## Abbreviations

COVID-19: Corona virus disease 2019
RTPCR: Reverse transcriptase polymerase chain reaction
NIBP: Non-invasive blood pressure
LCCA: Left common carotid artery
LVEF: Left ventricular ejection fraction
IBP: Invasive blood pressure

## References

[CR1] Ashur C, Conlon A, Eagle K, Bowman M (2020). Influenza infection and aortic dissection: a commentary on the association between a viral syndrome and major cardiac events in the context of the current COVID-19 pandemic. J Allergy Infect Dis.

[CR2] Clerkin KJ, Fried JA, Raikhelkar J (2020). COVID-19 and cardiovascular disease. Circulation.

[CR3] Erbel R, Alfonso F, Boileau C, Dirsch O, Eber B, Haverich A (2001). Diagnosis and management of aortic dissection: task force on aortic dissection, European society of cardiology. Eur Heart J.

[CR4] Fukuhara S, Rosati CM, El-Dalati S (2020). Acute type A aortic dissection during COVID-19 outbreak. Ann Thorac Surg.

[CR5] Gregory SH, Yalamuri SM, Bishawi M, Swaminathan M (2018). The perioperative management of ascending aortic dissection. Anesth Analg.

[CR6] Hebballi R, Swanevelder J (2009). Diagnosis and management of aortic dissection. Continuing Educa Anaesth Crit Care Pain.

[CR7] Hiratzka LF, Bakris GL, Beckman JA, at el. (2010). ACCF/AHA/AATS/ACR/ASA/SCA/SCAI/SIR/STS/SVM Guidelines for the diagnosis and management of patients with thoracic aortic disease. Circulation.

[CR8] Melvinsdottir IH, Lund SH, Agnarsson BA, Sigvaldason K, Gudbjartsson T, Geirsson A (2016). The incidence and mortality of acute thoracic aortic dissection: results from a whole nation study. European J Cardio-Thor Surg.

[CR9] Shah SB, Hariharan U, Chawla R (2021). Common anti-COVID-19 drugs and their anticipated interaction with anesthetic agents. J Anaesthesiol Clin Pharmacol.

[CR10] Tabaghi S, Akbarzadeh MA (2020). Acute type A aortic dissection in a patient with COVID-19. Future Cardiol.

[CR11] Totonchi Z, Givtaj N, Sakhaei M, Foroutan A, Chitsazan M, Chitsazan M (2015). Anesthetic management in a patient with type A aortic dissection and superior vena cava syndrome. Res Cardiovasc Med.

